# Icariin Protects Rat Cardiac H9c2 Cells from Apoptosis by Inhibiting Endoplasmic Reticulum Stress

**DOI:** 10.3390/ijms140917845

**Published:** 2013-08-30

**Authors:** Qiufang Zhang, Hongliang Li, Shanshan Wang, Ming Liu, Yibin Feng, Xuanbin Wang

**Affiliations:** 1 Laboratory of Chinese Herbal Pharmacology, Renmin Hospital, Hubei University of Medicine, Shiyan 442000, China; E-Mails: 20090581@hbmu.edu.cn (Q.Z.); 20090652@hbmu.edu.cn (H.L.); 20090600@hbmu.edu.cn (S.W.); 20090663@hbmu.edu.cn (M.L.); 2 Department of Pharmacology, Hubei University of Medicine, Shiyan 442000, China; 3 School of Chinese Medicine, Li Ka Shing Faculty, the University of Hong Kong, Hong Kong, China; E-Mail: yfeng@hku.hk; 4 School of Pharmacy, Hubei University of Medicine, Shiyan 442000, China

**Keywords:** icariin, endoplasmic reticulum stress, apoptosis, cardioprotection

## Abstract

Endoplasmic reticulum stress (ERS) is one of the mechanisms of apoptotic cell death. Inhibiting the apoptosis induced by ERS may be a novel therapeutic target in cardiovascular diseases. Icariin, a flavonoid isolated from *Epimedium brevicornum* Maxim, has been demonstrated to have cardiovascular protective effects, but its effects on ERS are unknown. In the present study, we focused on icariin and investigated whether it might protect the cardiac cell from apoptosis via inhibition of ERS. In H9c2 rat cardiomyoblast cells, pretreatment of icariin significantly inhibited cell apoptosis by tunicamycin, an ERS inducer. Icariin also decreased generation of reactive oxygen species (ROS), loss of mitochondrial membrane potential and activation of caspase-3. Moreover, icariin inhibited upregulation of endoplasmic reticulum markers, GRP78, GRP94 and CHOP, elicited by tunicamycin. These results indicated that icariin could protect H9c2 cardiomyoblast cells from ERS-mitochondrial apoptosis *in vitro*, the mechanisms may be associated with its inhibiting of GRP78, GRP94 and CHOP and decreasing ROS generation directly. It may be a potential agent for treating cardiovascular disease.

## 1. Introduction

Endoplasmic reticulum stress (ERS) is involved in cardiovascular pathological processes, such as atherosclerosis, ischemic heart disease, cardiomyopathy and heart failure. For example, in pressure overloaded heart failure, the ERS markers, glucose-regulated protein 78 (GRP78) and C/EBP homologous protein (CHOP) were increased [1]. Furthermore, in a transaortic constriction mice model, cardiac hypertrophy and dysfunction were attenuated in CHOP knockout mice compared with the wild type [2] In Akita diabetic mice, mRNA levels of GRP78/CHOP are increased, and deletion of CHOP delays the onset of diabetes. In these processes, ERS can lead to apoptosis. Apoptosis of cardiomyocytes results in the loss of contractile tissue, heart dysfunctions and the promotion of cardiac hypertrophy and reparative fibrosis, which all contribute to the development of cardiovascular diseases [3–5]. For the cell biological mechanisms, the endoplasmic reticulum (ER) is an organelle responsible for regulating protein synthesis, protein folding and trafficking and intracellular calcium levels [6,7]. Accumulation of misfolded or unfolded proteins in the ER can result in ERS or unfolded protein response (UPR). In the early stages of ERS, a series of signaling pathways are activated, and ER chaperone proteins, such as GRP78 and GRP94. are overexpressed to increase cell folding capacity and prevent cells from damage. However, excessive and prolonged ERS induces the overexpression of C/EBP homologous protein (CHOP) and promotes cell apoptosis [4,8–11]. ER stress is also involved in apoptosis by generation of endogenous reactive oxygen species (ROS) or causing a disturbance of Ca^2+^ homeostasis in mitochondria, leading to activation of caspase-3, an executor of caspase-dependent apoptosis [11]. Therefore, inhibiting the apoptosis induced by ERS may be a novel therapeutic target.

Icariin, a flavonoid isolated from the Chinese medicinal herb, *Epimedium brevicornum* Maxim (Berberidaceae. Chinese name: Yinyanghuo) [12], has been demonstrated to have a wide range of pharmacological and biological activities, including estrogenic activity, antioxidant effects, anti-tumor activity, immunoregulation, neuroprotection and improving sexual function [13–18]. Recent studies suggested that icariin facilitated the directional differentiation of embryonic stem cells into cardiomyocytes *in vitro* and possessed cardioprotective effects during hypoxia [19,20]. Furthermore, icariin significantly attenuated cardiomyocyte apoptosis and ameliorated left ventricular dysfunction and cardiac remodeling. The mechanism was associated with regulating Bcl-2/Bax expression, MMP-2 and MMP-9 [21]. However, its role in ERS and the underlying mechanisms have yet to have been studied. We hypothesized that the cardioprotective effects of icariin on cardiomyocyte are at least partially due to reducing ERS. The present study was to investigate whether icariin protects H9c2 rat cardiomyoblast cells from ERS inducer-mediated apoptosis by the ERS pathway.

## 2. Results

### 2.1. The Effects of Icariin on Cell Viability in H9c2 Cells

**Figure 1:**
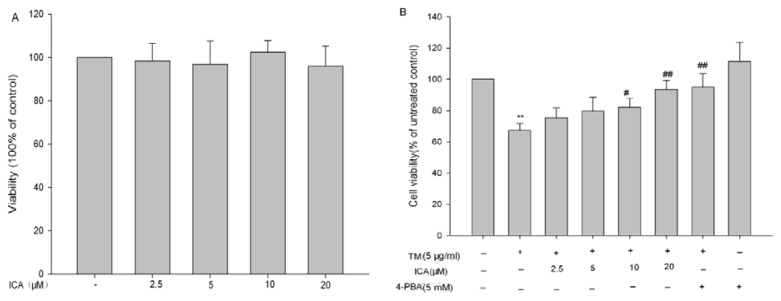
Effects of icariin and tunicamycin on H9c2 cells viabilities. (**A**) Effects of icariin on H9c2 cell viabilities. Cell viability was measured by the 3-(4,5-Dimethylthiazol-2-yl)- 2,5-diphenyltetrazolium bromide (MTT) assay after treatment of H9c2 cells with icariin for 24 h at indicated concentration; (**B**) Icariin dose-dependently protected H9c2 cells from tunicamycin-induced cytotoxicity. Cells were pretreated with icariin at different concentrations for 30 min, and cell viability was measured by the MTT assay after treatment with 5 μg/mL tunicamycin for 24 h. Five millimolar of 4-PBA served as a positive control. Data are shown as the means ± SEM of three independent experiments. **p < 0.01 *vs*. the untreated control group; ^#^*p <* 0.05, ^##^*p* < 0.01 *vs*. the tunicamycin group (TM = tunicamycin, ICA = icariin, 4-PBA = 4-phenylbutyrate).

To investigate the effect of icariin on cell viability, H9c2 cells were treated with 0, 2.5, 5, 10 and 20 μM icariin or 5 mM 4-phenylbutyrate (4-PBA, an ERS inhibitor) for 24 h. The results showed that none of them had a significant effect on the cell viabilities (Figure 1A). In contrast, after treatment with 5 μg/mL of tunicamycin (an ERS inducer), the viability of H9c2 cells was significantly decreased to 67.33% ± 4.48% (*p* < 0.01). However, the viability of H9c2 cells was increased up to 75.34% ± 6.23%, 79.72% ± 8.84%, 82.04% ± 6.03%, 93.51% ± 5.91% and 95.06% ± 8.50%, when pretreated with icariin (2.5, 5, 10 and 20 μM) or 4-PBA (5 mM) for 30 min, respectively (Figure 1B). Therefore, the results showed that icariin could protect H9c2 cells from tunicamycin-induced cell death in a concentration-dependent manner.

### 2.2. Icariin Reduces Apoptotic Cells Induced by Tunicamycin in H9c2 Cells

**Figure 2:**
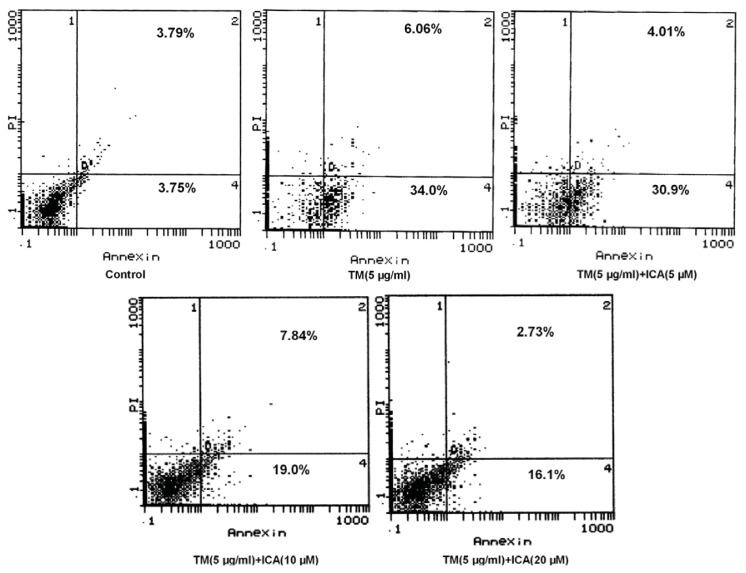
Effects of icariin on cell apoptosis induced by tunicamycin. H9c2 cells were pretreated with icariin (5, 10 and 20 μM) for 30 min and, then, treated with 5 μg/mL tunicamycin for 24 h. Cell apoptosis was measured by annexin V/PI staining (flow cytometric analyses). Cells that stained positive for annexin V and negative for PI were at the early apoptotic stage (Q4). Cells that stained positive for both annexin V and PI were at the end stages of apoptosis (Q2). The data shown is representative of the best of three individual fluorescence activated cell sorter (FACS) acquisitions (TM = tunicamycin, ICA = icariin).

**Figure 3:**
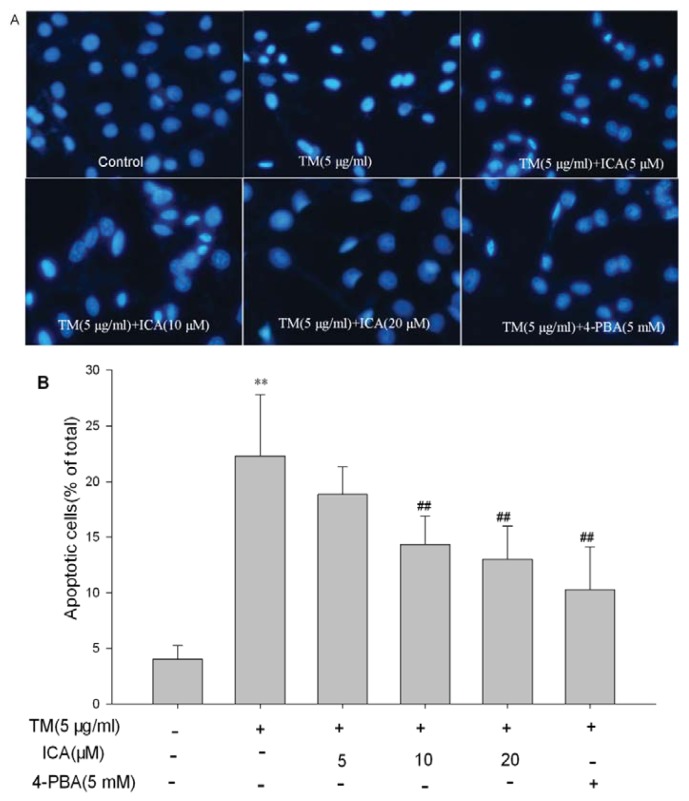
Icariin attenuated tunicamycin-induced apoptosis of H9c2 cells. H9c2 cells were pretreated with icariin (5, 10 and 20 μM) or 5 mM 4-PBA (an endoplasmic reticulum stress (ERS) inhibitor) for 30 min and, then, treated with 5 μg/mL tunicamycin for 24 h. (**A**) Hoechst 33258 staining was performed to visualize the extent of apoptotic cells. Condensed or fragmented nuclei were considered as apoptotic cells (magnification: 200×); (**B**) Quantification of the percent of apoptotic H9c2 cells after exposure to tunicamycin and icariin or 4-PBA. The content was calculated as the ratio of abnormal nuclei (condensation and fraction) to the total number of nuclei by Hoechst staining. All data are present as the mean ± SEM from triplicate independent experiments, ** *p* < 0.01 *vs*. the untreated control group; ^##^*p* < 0.01 *vs*. the TM group (TM, tunicamycin, ICA, icariin, 4-PBA, 4-phenylbutyrate).

In order to examine the effect of icariin on the cell apoptosis induced by tunicamycin, the annexin V/propidium iodide (PI) double-staining assay was used to stain H9c2 cell, and the apoptosis rate was analyzed by flow cytometry. As the data showed in the vehicle group, the basal early apoptotic rate was about 3.75%, and it was increased up to about 34.0% after treatment with 5 μg/mL tunicamycin for 24 h. However, when pretreated with icariin (5, 10 and 20 μM) for 30 min prior to tunicamycin, the early apoptosis rate was decreased to 30.9%, 19.0% and 16.1%, respectively (Figure 2). Hoechst 33258 staining was used to further investigate the anti-apoptotic effect of icariin. Similar results were obtained in H9c2 cells pretreated with ERS inhibitor 4-PBA (Figure 3).

**Figure 4:**
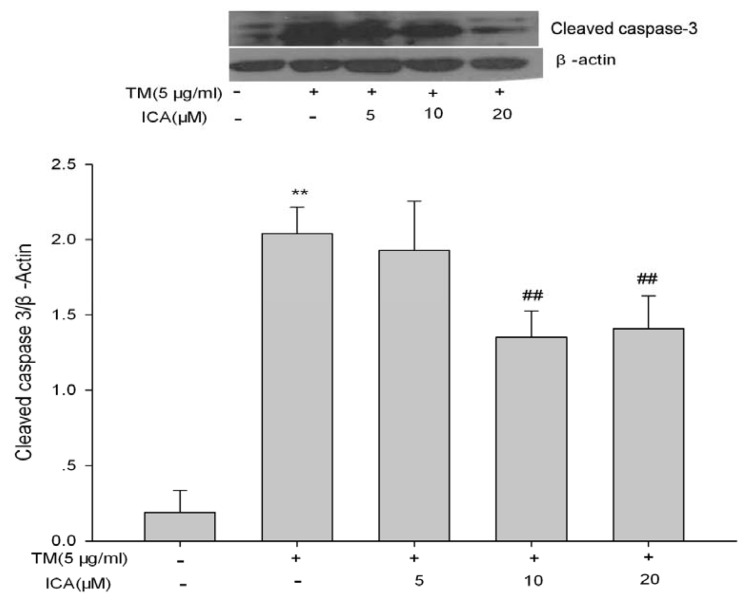
Icariin reduces cleaved caspase-3 expression induced by tunicamycin in H9c2 cells. H9c2 cells were pretreated with icariin (5, 10 and 20 μM) for 30 min and, then, treated with 5 μg/mL tunicamycin for 24 h. Cleaved caspase-3 Rabbit mAb was used to detect endogenous level of the large fragment (17/19 kDa) of activated caspase-3 by western blot. The intensity of each band was quantified by densitometry, and data were normalized using the intensity of β-actin. **p < 0.01 *vs*. the untreated control group; ^##^*p* < 0.01 *vs*. the TM group (TM = tunicamycin, ICA = icariin).

Caspase-3 is an executor of apoptosis and is involved in many important events that lead to the completion of apoptosis. Cleaved caspase-3 is a marker of caspase-3 activation. Compared to the control group in this study, tunicamycin significantly increased cleaved caspase-3 expression, and icariin at 10 μM and 20 μM significantly reduced this elevated level, though there is no statistical difference between the two groups of icariin at 10 μM and 20 μM (Figure 4).

### 2.3. Effects of Icariin on the Intracellular ROS Level in Tunicamycin-Treated Cells

**Figure 5:**
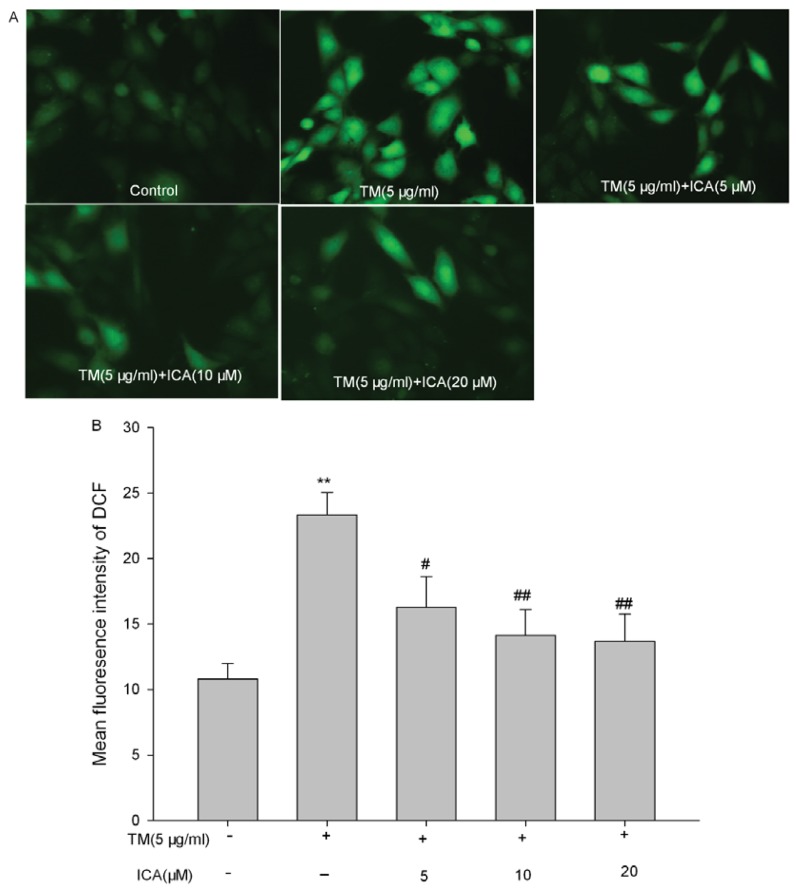
Effects of icariin on reactive oxygen species (ROS) generation induced by tunicamycin. Quiescent H9c2 cells were pretreated with icariin (5, 10, 20 μM) for 30 min and, then, treated with 5 μg/mL tunicamycin for 24 h. The intracellular ROS level was measured by the fluorescent probe (2′,7′,-dichlorofluorescein diacetate (DCFH-DA)). (**A**) The fluorescent images were obtained by fluorescence microscopy (magnification: 200×). The representative results from three independent experiments are shown; (**B**) Quantitative analysis of mean fluorescence intensity in each group was calculated by Image J 1.44 pro software (Media Cybernetics, Inc., Bethesda, MA, USA). The data represent the means ± SEM of three independent experiments, **p < 0.01 *vs*. the untreated control group; ^#^*p* < 0.05, ^##^*p* < 0.01 *vs*. the tunicamycin group (TM = tunicamycin, ICA = icariin).

Tunicamycin significantly increased the intracellular level of ROS. Pretreatment with (10 and 20 μM) icariin significantly inhibited the elevated intracellular concentration of ROS induced by tunicamycin (*p* < 0.05). Although icariin at the 5 μM could reduce ROS compared with the tunicamycin group, the data did not reach statistical significance (*p* > 0.05) (Figure 5).

### 2.4. The Effect of Icariin on ΔΨm in H9c2 Cells

**Figure 6:**
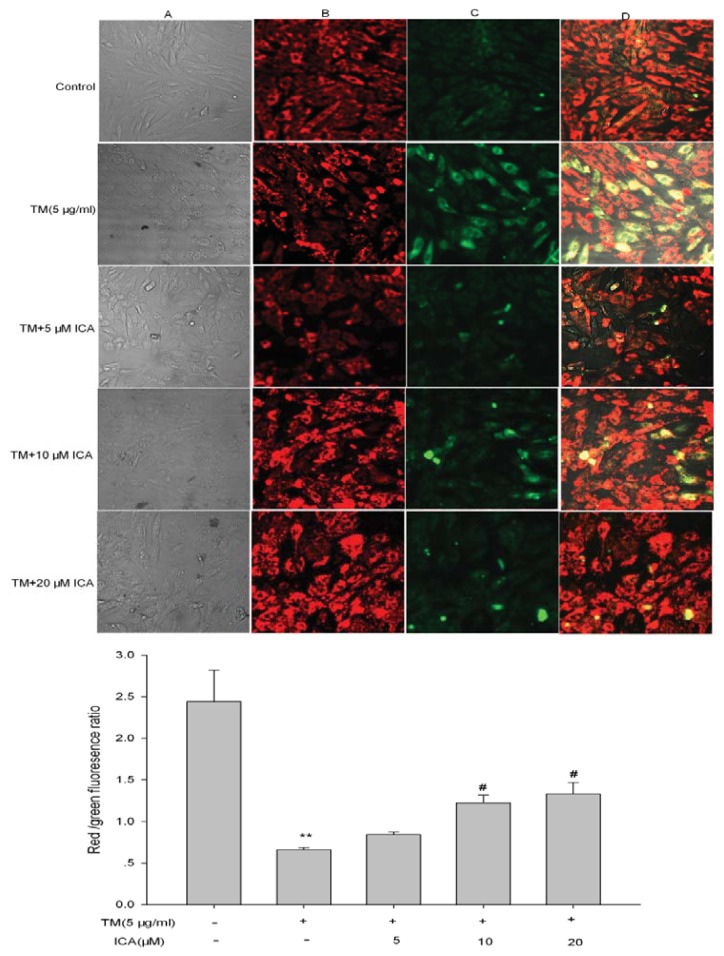
Icariin attenuates tunicamycin-induced mitochondrial membrane potential (ΔΨ) loss in H9c2 cells. Quiescent H9c2 cells were pretreated with icariin (5, 10, 20 μM) for 30 min and, then, treated with 5 μg/mL tunicamycin for 24 h. JC-1 fluorescence was measured by confocal microscopy to assess the emission shift from red (590 nm) to green (530 nm) using 488 nm excitation. (**A**) The light field of H9c2 cells after different treatment; (**B**) The image shows red JC-1 fluorescence. Red fluorescence represents large, negative ΔΨ; (**C**) The image shows green JC-1 fluorescence. Higher green fluorescence represents mitochondrial membrane potential loss; (**D**) The image shows red/green JC-1 fluorescence. Results are representative of three independent experiments. **p < 0.01 *vs*. the untreated control group; ^#^*p* < 0.05 *vs*. the TM group (TM, tunicamycin, ICA, icariin).

Static H9c2 cells stained with 5,5′,6,6′-tetrachloro-1,1′,3,3′-tetraethyl-imidacarbocyanine iodide (JC-1) presented red fluorescence with a little green fluorescence, indicative of a normal polarization state. Exposure of H9c2 cells to tunicamycin for 24 h resulted in dissipation of ΔΨm, which was shown as increased green fluorescence by JC-1 staining. Pretreatment with different concentrations of icariin could moderate the dissipation of ΔΨm, indicating the protective effect of icariin. The ratio of green and red fluorescence was used to demonstrate the toxicity of tunicamycin in mitochondria and the protective effect of icariin. In the vehicle group, JC-1 aggregated in mitochondria, and the ratio was 2.44 ± 0.38 and that of the tunicamycin group was 0.66 ± 0.03, while that of the groups pretreated with five, 10 and 20 μM of icariin was increased up to 0.83 ± 0.03, 1.22 ± 0.09 and 1.33 ± 0.14 (Figure 6).

### 2.5. The Effects of Icariin on ERS Marker Proteins

**Figure 7:**
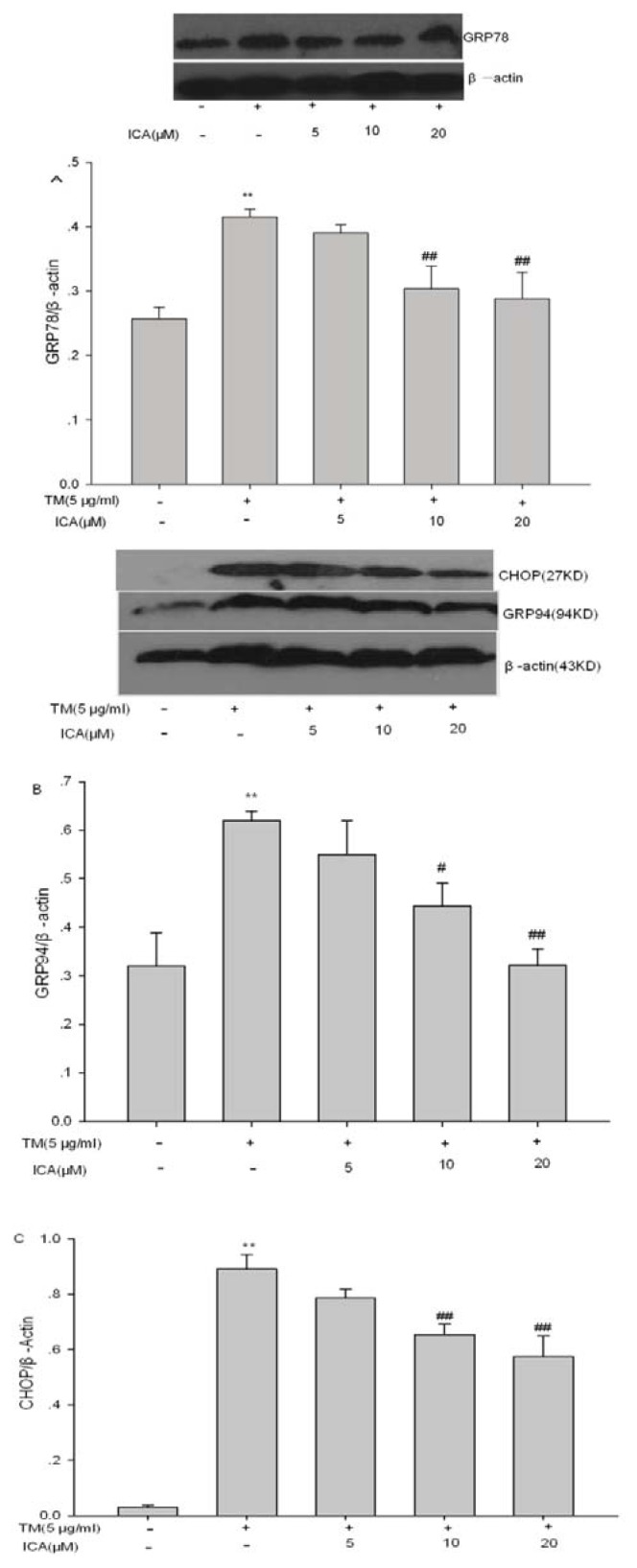
Icariin directly inhibits ERS induced by tunicamycin. ERS marker proteins, GRP78, GRP94 and CHOP, were detected by Western blot. Quantitative data of GRP78 (**A**); GRP94 (**B**) and CHOP (**C**) were given, and values represent the means ± SEM of three independent experiments. **p < 0.01 *vs*. the untreated control group; ^#^*p* < 0.05, ^##^*p* < 0.01 *vs*. the TM group (TM = tunicamycin, ICA = icariin).

To identify the direct effect of icariin on ERS, ERS marker proteins, GRP78, GRP94 and CHOP, were examined. Western blot assay showed that the protein levels of GRP78, GRP94 and CHOP were significantly increased with the presence of tunicamycin for 24 h. However, Icariin at 10 and 20 μM, but not 5 μM, significantly inhibited the expression of GRP78, GRP94 and CHOP induced by tunicamycin (Figure 7).

## 3. Discussion

According to the relevant reports [18,22,23], many unfolded proteins might occur in endoplasmic reticulum and trigger disruption of cell redox status, leading to apoptosis. Treated with the endoplasmic reticulum stressor, tunicamycin, for 24 h, cell apoptosis and ERS related-protein were detected. However, pretreatment of icariin significantly attenuated cardiomyocyte apoptosis and ameliorated left ventricular dysfunction and cardiac remodeling. Whether the mechanisms were associated with inhibiting ERS is unclear. In this study, we examined the effects of icariin on ERS and ERS-induced apoptosis. The underlying mechanisms in this process were also investigated.

ERS participates in the pathogenesis of various cardiovascular diseases and promotes disease progression [24]. A variety of extracellular stresses, such as nutrient deprivation, hypoxia, oxidative stress and calcium imbalance, led to unfolded protein response (UPR) in the ER. GRP78 and GRP94 are upregulated in the early stages of ERS, followed by overexpression of CHOP in the prolonged ERS. CHOP is accumulated in the nucleus and promotes cell apoptosis by downregulating Bcl-2, depleting glutathione, increasing the production of reactive oxygen species (ROS), causing the depolarization of the mitochondrial membrane potential and the release of cytochrome *c* in cardiomyocytes [9,10,25,26]. A growing body of evidence reported that antioxidants, such as metallothionein, Mnetrakis (1-methyl-4-pyridyl) porphyrin (MnTMPyP) and *N*-acetyl-cysteine (NAC), have been shown to protect the cardiac cell by inhibited ER stress. These suggested that antioxidants could reduce ERS-mediated apoptosis by decreasing ROS generation directly [22].

Icariin is a natural product from the Chinese medicine, *Epimedium brevicornum* Maxim (Berberidaceae), exhibiting a protective function in many cardiovascular diseases [19,27]. In the present study, we found that icariin inhibited the effects of tunicamycin (an ERS inducer) to increase the cell viability and decrease apoptosis in H9c2 cells.

To further identify the mechanisms of icariin on the protection of H9c2 cells, we used 4-PBA, which can inhibit ERS and block expression of GRP78 and CHOP [28,29]. The results showed that pretreatment with icariin decreased apoptosis and increased cell viability, mimicking the effects of 4-PBA pretreatment, suggesting that icariin protected H9c2 cell injuries by inhibiting ERS.

Data also showed that icariin attenuated ROS production and, subsequently, decreased mitochondrial membrane potential loss and caspase-3 cleavage. Previous studies showed that ERS-induced ROS production may be initiated by ER and pushed mitochondrial ROS above the threshold for cell survival, leading to cell death. Meanwhile, mitochondrial ROS can impair ER function and enhance ER stress [30]. Direct or indirect production of ROS can mediate cytochrome *c* release and trigger caspase activation. Icariin is a flavonoid and also has antioxidative effects on various pathological processes, such as stroke, ischemia reperfusion-induced cognitive impairments and congestive heart failure [14,15,20,27,31–33]. The results in this study suggested that besides reducing ROS by inhibiting ERS, icariin might simultaneously inhibit the mitochondria-mediated caspase signaling pathway by reducing ROS directly.

Concerning the effects on the markers of ERS, GRP78 and GRP94 are regarded as the biomarkers that represent the activation of ER stress. Meanwhile, CHOP is the first identified protein that plays an important role in ERS-induced apoptosis. Chop is a downstream component of ERS at the convergence of the IRE1, PERK and ATF6 pathways. As a key component of the final stage of ERS, CHOP leads to cell apoptosis or cycle arrest. Overexpression of CHOP promotes apoptosis, while deficiency of CHOP can prevent ERS-induced apoptosis [34]. Our findings showed that icariin inhibited not only CHOP, but also pro-survival proteins, GRP78 and GRP94, which suggested that icariin depressed the degree of ERS and potentiated the ability of H9c2 cells to survive from strong stress. However, how to inhibit GRP78 and CHOP upregulation by icariin still needs further study.

## 4. Experimental Section

### 4.1. Antibodies and Reagents

Tunicamycin(TM) and 4-phenylbutyrate (4-PBA) were purchased from Sigma (St. Louis, MO, USA). JC-1, DCFH-DA and annexin V/PI were obtained from Beyotime Institute of Biotechnology (Haimen, China). High glucose Dulbecco’s modified Eagle’s medium (DMEM) was a product of Gibco BRL (Gaithersburg, MD, USA). Fetal bovine serum was a product of Sijiqing Biological Engineering Materials Corp (Hangzhou, China). Icariin was purchased from National Standard Sample Centre (Beijing, China). Mouse monoclone anti-Cleaved caspase-3, rabbit monoclone anti-CHOP and rabbit monoclone anti-GRP94 were purchased from Cell signaling Technology (CST, Boston, MA, USA). Rabbit polyclonal anti-GRP78 was from Santa Cruz Biotechnology (Santa Cruz, CA, USA).

### 4.2. Cell Cultures

H9c2 cells, derived from embryonic heart tissue (American Type Culture Collection, Manassas, VA, USA) were cultured in high glucose (4500 mg/L) Dulbecco’s modified Eagle’s medium (DMEM) supplemented with 10% (*v*/*v*) fetal bovine serum, 100 U/mL penicillin and streptomycin in tissue culture flasks at 37 °C in a humidified atmosphere of 5% CO_2_. The cells were fed every 2–3 days and subcultured once they reached 70%–80% confluent. H9c2 cells were pretreated with various concentrations of icariin or 5 mM 4-PBA for 30 min [29,35], then were exposed to tunicamycin at 5 μg/mL to induce endoplasmic reticulum stress in the presence of either icariin or 4-PBA for 24 h.

### 4.3. MTT Assay for Cell Viability

The assay is based on the reduction of the tetrazolium salt, MTT, by active mitochondria to produce water-insoluble formazan salt. Cells were treated in 96-well plates; 10 μL MTT (final concentration 0.5 mg/mL) was added to each well, followed by incubation for 4 h at 37 °C. The medium of the culture was removed by aspiration, and the cells were washed twice with phosphate buffered saline (PBS). 100 μL of dimethyl sulfoxide (DMSO) was added to dissolve blue formazan in living cells. Absorbance was read at 570 nm with a microplate reader (Sunrise, Tecan, Germany). The cells incubated with control medium were considered to be 100% viable. Cell viability percentage = the OD value of each treated group/OD value of control group × 100%. The effective concentration of icariin chosen for further experiments was based on these MTT results.

### 4.4. Hoechst 33258 Staining

For the Hoechst 33258 assay, the H9c2 cells were seeded at a density of 1.5 × 10^3^/100 mm on sterile glass cover slips with poly-l-lysine in six well of plates and grown to 80% confluence. Cells were treated according to the methods described above. Subsequently, cells were fixed, washed twice with PBS and incubated with Hoechst 33258, according to the manufacturer’s instructions (Beyotime, Haimen, China). Changes in the nuclei of cells after Hoechst 33258 staining were observed under a fluorescence microscope (Olympus, Tokyo, Japan). Apoptosis was determined according to the appearance of fragmented or condensed nuclei.

### 4.5. Assessment of Apoptosis by Flow Cytometry

Apoptosis was identified by means of double fluorescence staining with annexin V/propidium iodide (PI). In apoptotic cells, the membrane phosphatidylserine was translocated from the inner to the outer surface of the plasma membrane, although the membrane remains physically intact. Apoptotic cells were, therefore, stained with annexin V-fluorescein isothiocyanate (FITC), which binds phosphatidylserine with high affinity, resulting in green fluorescence when excited at 620 nm. Necrotic cells lost the physical integrity of their plasma membrane and were, therefore, stained with both annexin V-FITC and PI [36]. Live cells were unstained. H9c2 cells (1 × 10^6^ cells per sample) were loaded with 5 mL PI and 10 mL annexin V-FITC (Jingmei Biotech, Shanghai, China) at room temperature for 10 min in the dark. Cell apoptosis was examined by flow cytometer (Becton-Dickinson, Mountain View, CA, USA) within 1 h.

### 4.6. Assay of Intracellular Reactive Oxygen Species (ROS)

Intracellular ROS was measured with the non-fluorescent probe, 2′,7′,-dichlorofluorescein diacetate (DCFH-DA). In the presence of ROS, DCFH was oxidized to highly fluorescent dichlorofluorescein (DCF), which is trapped inside the cells. H9c2 cells were seeded at a density of 1 × 10^5^ per milliliter in six well culture dishes. One day after seeding, cells were pretreated with icariin for 30 min and, then, treated with tunicamycin (5 μg/mL) for 24 h. The cells were washed with PBS three times, and 10 μM DCFH-DA was added to cultures. The cells were incubated for 20 min at 37 °C. According to the manufacturer’s instruction, DCF fluorescence was measured over the entire field of vision using a fluorescent microscope connected to an imaging system (Olympus, Tokyo, Japan). Mean fluorescence intensity from three random fields was analyzed using Image J 1.44 pro software (Media Cybernetics, Inc., Bethesda, MA, USA), and the mean fluorescence intensity was used to represent the amount of ROS [37].

### 4.7. Measurement of Mitochondrial Membrane Potential (ΔΨm) Using JC-1

JC-1 easily penetrates cells and mitochondria. A red fluorescent JC-1 probe exists as “J-aggregates” in the normal hyperpolarized mitochondria. However, at low membrane potentials, JC-1 exists in the monomeric form and stains cells green. The ratio of green/red JC-1 fluorescence only depends on the mitochondrial membrane potential. Briefly, H9c2 cells were grown in the 24-well plates, then incubated at 37 °C for 30 min with 5 mg/L JC-1 [38] and, then, washed three times with PBS and placed in the free serum medium to be examined immediately under a confocal laser microscope (Fluoview, Olympus, Tokyo, Japan). JC-1 fluorescence was measured to assess the emission shift from green (530 nm) to red (590 nm) using 488 nm excitation.

### 4.8. Western Blot Analysis of GRP78, GRP94, CHOP and Cleaved-Caspase 3

Cells were rinsed twice with ice-cold phosphate buffered saline and were lysed in lysis buffer (20 mM Tris-HCl, pH 7.5, 150 mM NaCl, 0.5% sodium deoxycholate, 1% NP-40, 0.1% SDS, 1 mM ethylene diamine tetraacetic acid (EDTA), 10 Mm NaF, 1 mM sodium orthovanadate, 10 μg/mL leupeptin, 10 mM PMSF) for 30 min at 4 °C. Cell lysates were then clarified by centrifugation 12,000× *g* for 10 min at 4 °C, and the supernatant was collected for protein analysis and Western blot. Protein concentration was determined by bicinchonininc acid (BCA) kit. Equivalent amounts (40 μg) of protein samples were loaded and separated on 12% sodium dodecylsulfate (SDS)-polyacrylamide gels, then transferred to a nitrocellulose membrane (Millipore, Billerica, MA, USA). Blots were blocked with 5% skim milk in Tris-buffered saline with 0.1% Tween 20 (TBST) for 1 h at room temperature (RT) and, then, incubated with a primary antibody of GRP78, GRP94, CHOP, cleaved caspase-3 and β-actin at 4 °C overnight. The membrane was washed three times with TBST, followed by incubation with the appropriate secondary antibody of anti-rabbit (mouse) immunoglobulin G conjugated to horseradish peroxidase at room temperature for 1 h. Using an enhanced chemiluminescence detection kit (Pierce), the proteins were exposed to X-ray film, and the band densities were analyzed using Image J software. The average of each band density was determined and expressed as a percentage of control.

### 4.9. Statistical Analysis

Data were represented as the mean ± SEM from at least three independent experiments. Two group comparison was used with an unpaired *t*-test, and as appropriate, multiple group comparisons were made by one way ANOVA and Tukey’s or Newman-Keuls *post hoc* tests; *p* < 0.05 was considered to be statistically significant.

## 5. Conclusions

In conclusion, we firstly found that icariin protected rat cardiac H9c2 cells from cell death by inhibiting ERS, which was associated with mitochondrial apoptosis. Its mechanisms may underlie downregulation of GRP78, GRP94, CHOP expression and decreasing ROS generation directly. Our findings provide evidence of the novel effects of icariin against cardiomyocyte apoptosis via the ERS-mitochondrial pathway. We suggest that icariin would warrant further investigation as a potential treatment for cardiovascular disease induced by ERS.
